# Retrospective Evaluation of Claw Lesions, Inflammatory Markers, and Outcome after Abomasal Rolling in Cattle with Left Displacement of the Abomasum

**DOI:** 10.3390/ani11061648

**Published:** 2021-06-01

**Authors:** Theresa Tschoner, Yury Zablotski, Melanie Feist

**Affiliations:** Clinic for Ruminants with Ambulatory and Herd Health Services at the Centre for Clinical Veterinary Medicine, LMU Munich, Sonnenstrasse 16, 85764 Oberschleißheim, Germany; Y.Zablotski@med.vetmed.uni-muenchen.de (Y.Z.); Melanie.Feist@lmu.de (M.F.)

**Keywords:** dairy cattle, lameness, laterality, oral therapy, abomasal rolling

## Abstract

**Simple Summary:**

Lameness is a major welfare problem in cattle and is often diagnosed concurrent to left displacement of the abomasum. Lame cattle prefer to lie on the affected leg, and laterality of lying may have an effect on the etiology of left displacement of the abomasum. The objective of this study was to investigate the possible association between laterality of claw lesion and presence of left displacement of the abomasum. For this study, the data of 252 cattle were analyzed retrospectively. Inclusion criteria were: a diagnosis of left displacement of the abomasum and diagnostic examination of claw lesions during hospitalization at the clinic. There was no association between laterality of claw lesion and left displacement of the abomasum. Cattle with a claw lesion in their right limbs were not diagnosed with left displacement of the abomasum more often than cattle with claw lesions in their left limbs. A total of 46.4% of cattle were diagnosed with at least one claw lesion. These results emphasize the importance of the prevention of claw lesions, especially in the post-partum period, to improve cattle welfare.

**Abstract:**

Lameness is often diagnosed in cattle with left displacement of the abomasum (LDA). Laterality of lying has an effect on the etiology of LDA, and lame cows prefer to lie on the affected limb. The objective of this study was to investigate the possible association between laterality of claw lesion and presence of LDA. The medical records of 252 cattle presented with a diagnosis of LDA and subjected to a diagnostic examination of claw lesions over a period of 11 years (2009–2019), were analyzed retrospectively. Data were evaluated for presence and localization of claw lesion, concentrations of inflammatory markers, and abomasal rolling as therapy. At least one claw lesion was diagnosed in 46.4% of cattle. There was no association between laterality of claw lesion and LDA. Presence of claw lesion or oral drench and/or analgesic treatment did not have an effect on occurrence of relapse. A high number of cattle was diagnosed with both LDA and claw lesions. Focus should lie on preventing painful claw lesions in the dry and the early post-partum period. The high recurrence rate after abomasal rolling suggests that abomasal rolling should only be considered as a therapy for temporary relief, and surgical procedures should be discussed with farmers.

## 1. Introduction

Left displacement of the abomasum (LDA) is a common disease in high producing dairy cattle in early lactation [1,2]. The disease has a multifactorial nature with a lactation incidence of 0.05 to 5.0%. Occurrence of LDA is a result of two pathogenic pathways, hypomotility of the abomasum and accumulation of gas [2]. Those pathogenic pathways are results of different etiological factors, such as negative energy balance, reduction of feed intake, and blood calcium levels, among others [2,3]. Hypomotility as well as the accumulation of gas can result in the buoyancy of the abomasum [2]. Negative energy balance and concurrent diseases such as hypocalcemia, puerperal metritis or endometritis, and claw disorders (associated with lameness), are common in cattle with LDA [2,4,5]. According to previous studies, 10% [6] to 26.8% of cattle [4] with LDA are also diagnosed with lameness, which results in poorer overall recovery rates [4,7]. The estimated genetic correlation between displacement of the abomasum (DA) and lameness is 0.07 ± 0.14 [8]. Lameness is thought to be a risk factor for displacement of the abomasum as well as for ketosis [9], as lameness results in reduction in feeding times and a change in feeding behavior [10,11]. Inflammatory markers (such as haptoglobin) are elevated in lame cows [12,13] and previous data indicates an activation of the immune system [13] and a systemic acute phase response [12].

In lame cows, lying time and number of lying bouts increases [14,15]. Daily lying time, frequency/number of lying bouts [14,15,16], average duration of lying bouts [14], and side of recumbency (laterality) [15,16] can be assessed using electronic data loggers [14,15,16]. Lameness [15] as well as side of hock lesion [16] do not seem to have an influence on the side cows preferred to lie on. However, other authors found that lame cows prefer to lie on the affected limb after painful claw surgeries [17].

One of the signs described in cattle with LDA, among others, is lying on the animal’s right side to avoid the pain and pressure of the distending abomasum [18]. The side on which animals are lying seems to have an effect on the etiology of LDA [19]. It was shown that the outcome of abomasal rolling in a cow with LDA was improved by forcing the cow to only lie on her left side for two weeks following abomasal rolling [20]. Additionally, after roll-and-toggle pin suture procedure, prognosis for recovery was believed to be good when cows lay down on their left sides after surgery [18].

It is the authors’ observation that cattle admitted to the Clinic for Ruminants diagnosed with LDA are often also diagnosed with a claw lesion, mostly in one hind leg. The hypothesis of the present, retrospective study was that in cattle both diagnosed with LDA and concurrent claw lesions, claw lesions might be found more often in the right side than the left.

Therefore, the main objective of the current study was (1) to assess the frequency and laterality of claw lesions in cattle diagnosed with LDA. Further objectives were (2) to describe the outcome and recurrence rate of LDA in cattle with or without claw lesions which were treated by abomasal rolling, (3) to assess the concentrations of inflammatory markers in cattle diagnosed with LDA and none or at least one claw lesion, and (4) to assess the correlation of occurrence of lameness with time of last claw trimming, housing, and inflammatory markers.

## 2. Materials and Methods

### 2.1. Animals

For this study, the medical records of all cows (≥2 years and ≥first lactation) that were admitted to the Clinic for Ruminants with Ambulatory and Herd Health Services, LMU Munich, between the 1 January 2009 and the 31 December 2019 were analyzed retrospectively. The medical records of 713 animals with a diagnosis of LDA were identified using logbooks and the clinic’s electronic database. Out of these 713 cattle, animals were further selected for the present study if diagnostic examination of claw lesions (DECL) was performed at the clinic. These two inclusion criteria (LDA and DECL) were met by 252 out of 713 animals.

### 2.2. Review of Medical Records

Information that was retrieved from the medical records included signalment of the cow (age and breed), anamnesis (calving date and days post-partum, type of housing, time of the last claw trimming at home), and results of biochemical analyses for Sandholm’s glutaraldehyde test [21], blood concentrations of betahydroxybutyrate (BHB), total protein, albumin, globulin, and leucocyte count. Results of glutaraldehyde test were grouped as ‘≤3’, ‘>3 to ≤8’, ‘>8 to 16’, and ‘>16’ [22].

Diagnosis of postpartum metritis, subclinical ketosis (defined as BHB concentration from 1.2 to 2.99 mmol/L, [23]), and clinical ketosis (BHB > 2.99 mml/L, [23]) was recorded.

Additionally, timing of DECL was recorded. All cattle were grouped according to their diagnoses as either being diagnosed with LDA without a claw lesion (LDA − CLAW), or as LDA with an additional diagnosis of at least one claw lesion (LDA + CLAW).

Surgical procedures (omentopexy or endoscopic abomasopexy as described by Janowitz [24]) or abomasal rolling (defined as casting of cattle on a hydraulic tilt table in lateral recumbency, with or without DECL) were evaluated as treatment of LDA.

For animals treated by abomasal rolling, analgesic or oral therapy (oral drench) administered after abomasal rolling, as well as outcome, were recorded.

Due to the retrospective nature of the study, description of why and how animals were selected for either therapy by abomasal rolling and/or surgical correction of LDA, as well as for oral and/or analgesic treatment (and the drugs selected) cannot be provided, as this information are not given in the medical records of animals.

### 2.3. Review of Orthopedic Status

Time of last claw trimming at home as stated by farmers during anamnesis was grouped as ‘<6 months’, ‘6 to 12 months’, ‘>12 months’, or ‘never’. Records of clinical examination at the day of admission of all animals were reviewed for presence of an arched back (‘yes’ or ‘no’) and/or presence of lameness (‘yes’, ‘shuffling with both hind limbs’, or ‘no’). Lameness was evaluated while the animal was moving as well as standing. As the scoring system veterinarians used over the years was not indicated in the medical data, lameness was defined as either present (‘yes’) or not (‘no’). ‘Shuffling with both hind limbs’ was defined as animals repeatedly changing weight from one hind limb to the other.

DECL was always performed with the animal in lateral recumbency, using a hydraulic tilt table. Diagnosis of claw lesion was recorded for each claw and in total for every individual animal. If DECL or changing of bandages was done following surgery (either endoscopy as described by Janowitz [24] or omentopexy), cattle were put into lateral recumbency, lying on the side of the sound limb. If animals were put into left lateral recumbency, the abdominal belt was strapped behind the sutures, to prevent wound dehiscence.

### 2.4. Outcome of Therapy

A positive outcome (=survival) was defined as discharge from the hospital alive and not directly to a slaughterhouse. Cattle with negative outcome (=non-survival) were either euthanized due to fatal clinical and/or intraoperative findings pre-, intra-, or postoperatively or died in the clinic.

### 2.5. Statistical Analysis

Data analysis was performed using R 3.6.3 (2020-2-29, R Foundation for Statistical Computing, Vienna, Austria). Results with a *p*-value < 0.05 were considered statistically significant.

One sample chi-squared goodness-of-fit test was used for analysis of frequency of each limb and pathologies in each limb.

Two samples Pearson’s chi-squared test was used for analysis of frequency of claw lesions in total and bilateral, diagnosis of clinical parameters (presence of lameness or an arched back), and for the evaluation of the effect of oral therapy and/or analgesic treatment on recurrence of LDA after abomasal rolling.

Differences between animals’ age, days post-partum, and laboratory parameters were assessed with either a t-test for normally distributed data, or a Mann–Whitney U Test for not-normally distributed data.

A multiple logistic regression model was used to analyze the correlation of housing, time interval since the last claw trimming, and concentrations of BHB, total protein, and glutaraldehyde test, with the occurrence of claw lesions (as ‘yes’ or ‘no’). For these parameters, a total of 45 observations were excluded from the multiple logistic regression model due to the missing of data.

## 3. Results

### 3.1. Animals

Breeds included German Simmental (67.1%, *n* = 169), Holstein (24.2%, *n* = 61), Red Holstein (1.6%, *n* = 4), Brown Swiss (1.9%, *n* = 5), German Red cattle (0.4%, *n* = 1), and dairy-cross (4.4%, *n* = 11). In one animal (0.4%), breed was given as ‘unknown’. Distribution of mean age (in years) and days post-partum is given in Table 1.

### 3.2. Occurrence of Claw Lesions, Localization, and Cause of Claw Lesions

Claw lesions were diagnosed in 46.4% (*n* = 117) of 252 animals with LDA. There was no significant difference in number of claw lesions between LDA – CLAW and LDA + CLAW.

**Figure 1:**
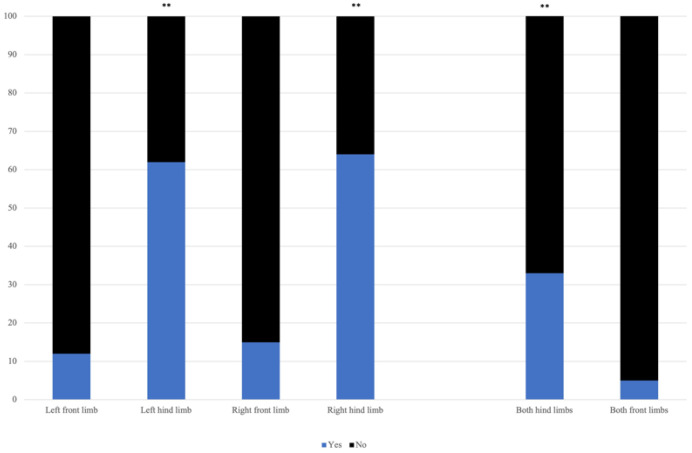
Distribution of occurrence of lameness in the front and hind limbs in animals diagnosed with left displaced abomasum and claw diseases (LDA + CLAW). Occurrence of claw lesions is given as ‘yes’ (blue) and ‘no’ (black). Significant differences of >0.01 are presented as **. Hind limbs were affected significantly more often than front limbs. In 33.3% of animals (*n* = 33), claw lesions were found in both hind limbs.

Claw lesions were found significantly more often (*p* < 0.01) in the hind limbs than in the front limbs. Distribution of percentage of affected limbs is presented in Figure 1. There was no significant difference in laterality of claw lesions. A total of 33.3% (*n* = 39) of animals was diagnosed with claw lesions in both hind limbs, and 4% (*n* = 5) with claw lesions in both front limbs. Claw lesions were found significantly more often (*p* < 0.01) in both hind limbs compared with both front limbs.

**Figure 2:**
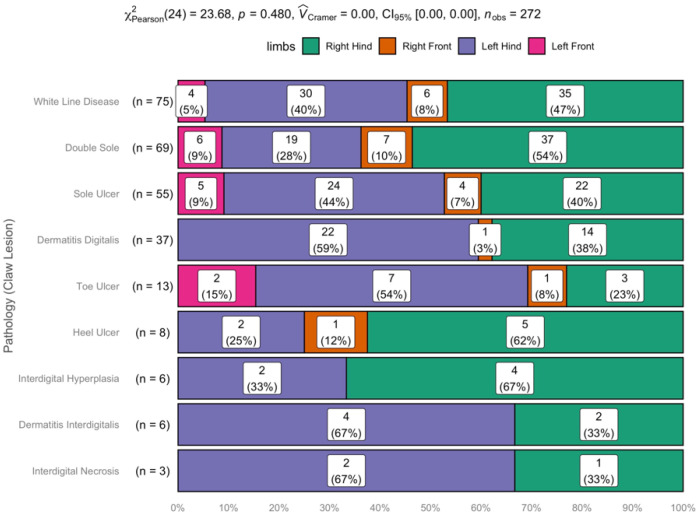
Occurrence of claw lesions according to pathologies in cattle diagnosed with left displacement of the abomasum (LDA) and claw lesions (LDA + CLAW). A total of 272 claw diseases was diagnosed in 117 cattle. Pathologies are given as percentage (%) and numbers in each limb. There was no significant association between particular claw lesions and a particular limb. X^2^_Pearson_ stands for Pearson’s Chi-square statistics and V_cramer_ for the effect size of Pearson’s Chi-square test. CI_95%_ are the 95% confidence intervals for V_cramer_. n_obs_ stands for the number of observations.

In total, 272 claw lesions were diagnosed in 117 cattle. Distribution of pathologies in total and for each limb is given in Figure 2. There was no significant association between particular claw lesions and a particular limb (*p* = 0.480).

### 3.3. Timing of Diagnostic Examination of Claw Lesions

DECL was always performed with the animal cast on a hydraulic tilt table in lateral recumbency. DECL was performed on day 1 ± 1.6 (0 to 9 days) of the animal’s stay at the clinic. Timing of DECL was not recorded for two animals.

In 29 animals, DECL was done following endoscopy as described by Janowitz [24] with the animals still in lateral recumbency. In 10 animals, DECL was performed 2 to 9 days after endoscopy as described by Janowitz [24] (day 4.8 ± 2.6 days), and in four animals 4 to days after omentopexy (5.0 ± 1.4 days). In all other animals submitted to surgery, surgery was performed 0 to 14 days after DECL (1.8 ± 1.8 days).

### 3.4. Treatment of Complicated Claw Lesions and Changing of Bandages

In 17 animals, complicated claw lesions (involving structures deeper than the corium) were found. Out of these animals, seven were euthanized due to the poor prognosis. In 10 animals, claw surgery was performed at day 4.7 ± 2.7 (1 to 10 days) of their stay at the clinic (*n* = 8 for amputation and *n* = 2 for resection of the bone). LDA was treated by abomasal rolling in 4 animals, by abomasal rolling followed by surgery in two animals, and by surgery in seven animals (*n* = 4 for endoscopic fixation as described by Janowitz [24], and *n* = 3 for omentopexy).

In 78 animals, changing of bandages was necessary during their stay at the clinic. Of these, 13 animals had not been submitted to surgery, 32 to endoscopic fixation as described by Janowitz [24], and 33 to omentopexy (*n* = 29 for omentopexy according to Dirksen [25], and two each for omentopexy and pyloropexy, respectively). Handling of changing of bandages was done as described for DECL following surgery. Wound dehiscence was seen in none of these animals following lateral recumbency on the hydraulic tilt table.

### 3.5. Laboratory Analysis and Concurrent Diseases

Results of laboratory analyses of BHB, glutaraldehyde test, total protein, albumin, globulin, and leucocyte count are given in Table 2.

Distribution of concurrent diseases both for LDA − CLAW and LDA + CLAW is given in Table 3.

### 3.6. Time of Last Claw Trimming and Lameness Examination

**Figure 3:**
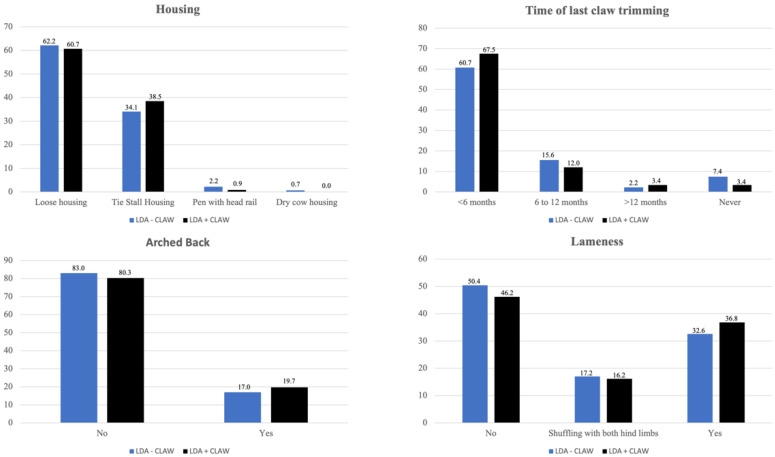
Housing, time of last claw trimming, and occurrence of arched back and lameness in 135 cattle diagnosed with left displaced abomasum without claw lesion (LDA − CLAW, blue), and 117 cattle diagnosed with left displaced abomasum and at least one claw lesion (LDA + CLAW, black). Number of cattle is given as %. Data missing for housing (*n* = 1 for LDA − CLAW) and for time of last claw trimming (*n* = 19 for LDA − CLAW and *n* = 35 for LDA + CLAW, respectively) is not presented as bars.

Age distribution (given as mean age in years) of the animals grouped according to the time of the last functional claw trimming at home is given in Table 4. Distribution of housing, time of last claw trimming, occurrence of an arched back, and lameness is given in Figure 3. Significant differences for lameness and posture within LDA − CLAW and LDA + CLAW are presented in Table 5. There were no significant differences in occurrence of an arched back or lameness between LDA − CLAW and LDA + CLAW.

### 3.7. Influence of Parameters on Claw Lesions

Out of 252 animals, a total of 45 observations were excluded from the multiple logistic regression model due to the missing of data. There was no association between BHB, glutaraldehyde test, total protein, or housing, and the occurrence of claw lesions. There was an effect of time since last claw trimming on claw lesions. Cattle in which no claw trimming had occurred (‘never’) showed a significantly (*p* < 0.04) lower risk for claw lesions compared with animals with claw trimming ‘<6 months’. For animals trimmed during the last ‘6 to 12 months’, there was a trend (*p* = 0.07) for claw lesions to occur less compared with animals trimmed ‘<6 months’.

### 3.8. Effect of Claw Lesion and Therapy on Outcome after Abomasal Rolling

Therapy consisted of abomasal rolling in 169 animals. In 3.0% (*n* = 5) animals, there was no information about outcome of abomasal rolling. Abomasal rolling was successful in 34.1% (*n* = 56) of all animals. Relapse occurred in 60.4% (*n* = 99) of animals after 0 to 13 (1.8 ± 1.9) days. In 5.5% (*n* = 9) of animals, abomasal rolling did not result in any movement of the abomasum (‘unsuccessful”, *n* = 5 for LDA − CLAW and *n* = 4 for LDA + CLAW).

In 156 animals, abomasal rolling by casting of the animal on a hydraulic tilt table in lateral recumbency was performed in combination with claw trimming. In 13 animals, abomasal rolling as described above was performed independently from claw trimming, either before (*n* = 6) or after (*n* = 7) the day of abomasal rolling.

Effect of claw lesions and therapy (analgesic and/or oral treatment) on relapse of LDA after abomasal rolling was analyzed for 164 animals with information about whether a relapse occurred. There was no significant effect of claw lesion on relapse (*p* = 0.459, Table 6). In all animals treated by abomasal rolling, significantly more animals suffered from a recurrence of LDA. There was no effect of oral and/or analgesic treatment on occurrence of relapse (*p* = 0.979, Table 6).

Analgesic therapy consisted of flunixine meglumine (*n* = 75), ketoprofen (*n* = 39), meloxicam (*n* = 23), metamizole (*n* = 11), and/or dexamethasone (*n* = 1). Components of oral therapy were vitamin E/selenium (*n* = 57), propylene glycol (*n* = 46), KCl (*n* = 60), calcium (*n* = 37), NadPh (*n* = 7), NaBic (*n* = 5), and/or MgO (*n* = 1). A list of analgesics administered to cows, and components used for the oral drench for each animal is given in Table S1.

### 3.9. Treatment by Surgery

In total, 198 animals was treated by surgery.

Surgery was performed in 66 animals without prior abomasal rolling. Omentopexy as described by Dirksen [25] was performed in 19.7% (*n* = 13) of animals, with the abomasum being in situ in 6 animals, and dislocated to the right in one animal. Pyloropexy was performed in 3.0% (*n* = 2) of animals, and endoscopy as described by Janowitz [24] in 77.3% of animals (*n* = 51).

In 132 animals which were first treated by abomasal rolling, either endoscopy as described by Janowitz [24] (39.4% (*n* = 52), 2 of these were 2 aborted and finished by omentopexy) or omentopexy (57.6% (*n* = 76), 2 of these were aborted and animals were euthanized) was performed. In these animals, omentopexy as described by Dirksen [25] was performed in 89.5% (*n* = 68) of animals, and omentopexy and pyloropexy each in 5.3% (*n* = 4) of animals. In 28 of the animals submitted to omentopexy following abomasal rolling, the abomasum was in situ.

### 3.10. Overall Outcome

Length of stay of animals at the clinic ranged from 0 to 29 (7.3 ± 4.7) days, with 6.4 ± 4.2 days for LDA − CLAW and 8.5 ± 4.8 for LDA + CLAW. In total, 82.9% (*n* = 209) of cattle were discharged to go home after 0 to 29 (7.9 ± 4.6) days. Out of 252 animals, 17.1% (*n* = 43) were euthanized after 0 to 21 (4.1 ± 3.9) days due to fatal clinical and/or intraoperative findings. Most common findings during necropsy were endometritis (*n* = 19), fatty liver disease (*n* = 16), local peritonitis (*n* = 6) and generalized peritonitis (*n* = 3) (following either perforated abomasal ulceration (*n* = 5), uterine perforation (*n* = 1), or cecal perforation (*n* = 1)), as well as mastitis (*n* = 5), bleeding abomasal ulcers (*n* = 3), arthritis (*n* = 8), phlegmon of a limb (*n* = 7), and decubitus (*n* = 5).

## 4. Discussion

This retrospective study was conducted to investigate a possible association between the laterality of claw lesions and the occurrence of LDA. According to our data, there was no difference in laterality of claw lesions in cattle diagnosed with LDA. At least one claw lesion was diagnosed in a high number of cattle with LDA.

In our study, 46.4% of cows with LDA were diagnosed with at least one claw lesion. This number is higher than the prevalence of 10% [6] to 26.8% [4] of cattle diagnosed with both LDA and claw lesions described in previous studies. The authors of both of these studies did not explain how assessment of lameness was performed. Therefore, it might be possible that the number of animals defined as ‘lame’ is higher in our study as we did not exclusively perform lameness scoring, but also diagnostic examination of the claws. The time difference between publication of the studies could also have resulted in these different findings. The aforementioned studies were published in 2004 [4] and 2008 [6]; in recent years, the incidence of lameness in dairy herds has increased greatly [26]. Additionally, animals admitted to our clinic were preselected by farmers’ veterinarians, possibly resulting in our study population consisting of moribund animals, which could explain the higher prevalence of lameness in our study.

Lameness is a serious problem for welfare and production in dairy cattle [14,15]. Lame cows spend less time on their feet [27], and 1.6 h longer lying in deep-bedded stalls [14]. Studies suggested that the laterality of lying has an effect on the etiology of LDA [18,20]. Therefore, it is reasonable to think that claw lesions in the right front or hind limb, and therefore increased lying on the right side, could facilitate the development of LDA. However, our data show that there was no significant difference in laterality and localization of claw lesions in cattle diagnosed with LDA and at least one claw lesion. More than one third of animals in our study population were diagnosed with claw lesions in not only one but both hind limbs. This could have resulted in animals lying on both their right or left side, and therefore might have affected our results. According to Feist et al. [17], cattle tend and prefer to lie on the side of the affected limb after claw surgeries. On the other hand, studies showed that there is no difference in laterality of lying between lame and non-lame cows [15], or in cows with hock lesions of different severity [16]. Looking at these contradictory results from previous studies, lameness laterality does not seem to be trustworthy as a proxy for lying behavior. Additionally, the presented data were evaluated retrospectively; therefore, we did not monitor lying behavior with cameras or pedometers and cannot make a statement about laterality of lying in our study population, which is the major limitation of this study. Therefore, a prospective study about laterality of lying in cattle with claw diseases and possible development of LDA would be advisable.

The claws of the hind limbs were affected more often by claw lesions than the claws of the front limbs. This agrees with other studies [28], and can be explained by the hind limbs carrying more weight [29].

According to our data, the most common claw lesions in cattle were white line disease (27.6%), double sole (25.4%), and sole ulcer (20.2%). This is not in accordance with previous studies. In Swiss dairy cows, prevalence for white line disease, double sole, and sole ulcer was 4.8%, 2.6%, and 11.5%, respectively [30]. In Austrian heifers, the prevalence of claw lesions was 87.1% for white line disease, 47.5% for double sole, and 1.7% for sole ulcer [31]. However, our study population consisted only of cattle in the first 60 to 100 days post-partum. Claw lesions mostly develop in this post-partum period, and are associated with metabolic disorders, which are common around the time of calving [32], as well as with management of cows. Optimizing claw health management includes, e.g., claw trimming at drying off and during early lactation (60 to 100 days in milk), locomotion scoring every two weeks and management of fresh cows [33]. Due to the loss of body condition and fat of the digital cushion [30], and the failure of the suspensory system of the third phalanx around calving [34], the high proportion of sole ulcers in our study can be explained. A high prevalence of lameness in cattle with a low BCS was also presented in previous works [35]. The thickness of the digital cushion is significantly associated with the incidence of sole ulcers and white line disease [36]. As body condition score of cattle was not recorded in our data, and we have no information about the (possible) loss of body condition after calving, we cannot be sure about the influence of previous body condition dynamics on claw lesions in our study. Another factor resulting in this high number of sole ulcers in our study population might be that in 27.6% of cattle, the last claw trimming was not performed in >6 to 12 months. It is recommended that claw trimming is performed 2 to 3 times per year, and in the first 60 to 100 days post-partum [33]. Age of the animals should be considered as well. With increasing age, laminitis-related claw lesions increase as well [37], resulting in older cows showing higher risk for sole ulcers. Sole ulcers are considered one of the most severe claw lesions in cattle [13]. Sole ulcer, double sole, and interdigital phlegmon are linked with increasingly poor locomotion [38]. Sole damage also results in 1.1 times more lying bouts in housed cows and cows kept on pasture [39]. The claw pathologies most often found in our study population were white line disease, double sole, and sole ulcer. Therefore, it is possible that these painful claw pathologies might have affected cows’ lying behavior more than less painful claw pathologies, e.g., an interdigital hyperplasia. Again, as we were not able to observe lying behavior in our animal population due to the retrospective nature of the study, we cannot make a statement about the influence of the claw pathologies on lying behavior.

Cattle, which had never been submitted to claw trimming, showed a significantly lower risk for claw lesions, compared with animals that had been trimmed in the previous 6 months. This is not in accordance with other research. The prevalence of sole ulcers was twice as high in cattle trimmed only once, compared with twice a year [40]. In our study, cattle in which no claw trimming had ever been performed were 2.8 ± 1.4 years old, as opposed to cattle which were submitted to a claw trimming in the previous 6 months, which were 5.4 ± 1.7 years old. In adult cattle as well as in heifers, routine foot trimming should be performed. Prophylactic foot trimming around the time of first calving is important [41], and is recommended around first insemination or at least two months [31] to 60 days prior to expected calving [33]. In cows, claw trimming around drying off results in 20% lower odds of sole ulcers in the following lactation [42]. Prevalence of lame cows and sole ulcers at claw trimming in spring is reduced by trimming 4.5 months earlier [40].

As the prevalence of laminitis-related claw lesions is higher with increasing age [37], the age difference between groups could have resulted in these significant differences, and explain why animals in LDA − CLAW were significantly younger than animals in LDA + CLAW. However, not only the timing of claw trimming, but also other factors influencing lameness, such as housing [35,43], nutrition and management of cow groups [33], or risk factors at cow level [44,45] should be considered, as these other variables might explain the lower prevalence of claw lesions in cattle never submitted to claw trimming. However, due to the retrospective nature of our study, these other factors were not provided and could therefore not considered for our model.

According to our data, inflammatory markers (glutaraldehyde test and blood concentrations of total protein) tended to be higher, with globulin being significantly higher, in LDA + CLAW compared with LDA − CLAW. This is in accordance with previous studies. Inflammatory markers, such as haptoglobin, are higher in cattle with claw lesions compared with sound animals [12,13]. Leucocyte counts in our study population did not differ between lame and non-lame cattle, which was already published in 2015 [13]. However, more than one third of cattle in both groups was diagnosed with postpartum metritis. Clinical metritis results in higher circulating concentrations of inflammatory markers, e.g., haptoglobin [46,47], which could have affected laboratory results.

The benefits of different surgical techniques for the correction of LDA are discussed elsewhere [48,49]. Due to the retrospective nature of our paper, we cannot offer an explanation about why certain surgical techniques were used. Factors for choosing a certain technique might include direction of displacement, the possible presence of adhesions, or surgical correction of a prior displacement; in the end, the technique chosen mostly depends on the preference of the surgeon [50].

Abomasal rolling, as a therapy for LDA, can result in abomasal volvulus, and recurrence rates are described to be between 50 and 70% [51]. This is in accordance with our results. However, percentage of abomasal rolling being unsuccessful was higher in our study, compared with previous work (5.5 compared with 1.5%, [52], respectively). The patient population in our study was possibly preselected by the referring veterinarians, resulting in a study population with a high number of concurrent diseases, as ‘easy’ cases are often treated in the field. This could have resulted in our lower success rate for abomasal rolling. It is also possible that the procedure of abomasal rolling is not successful because of adhesions between the abomasum and the body wall [52]. Lombar and Zadnik (2010) stated that LDA in 58% of cattle could be successfully treated by abomasal rolling, followed by 50 L of an oral electrolyte therapy [53]. In our study, 60.4% of animals treated by abomasal rolling did show a relapse of LDA, even though rolled animals received oral fluid and/or electrolyte therapy. However, as our data were not obtained from a prospective study, therapy after abomasal rolling was inconsistent and not all animals were treated with either an oral drench, an NSAID, or both, and results with other studies cannot be compared easily. As relapse can occur up to several weeks [51], it is possible that the recurrence rate in our patients was even higher, but was not diagnosed as animals were discharged to go home, or surgery was performed in animals with the abomasum being in situ.

Cure rates after omentopexy are decreased in cattle with lameness [7]. Our data indicate that presence of claw lesions did not have an effect on the occurrence of relapse (and therefore cure rates) after abomasal rolling. This is in accordance with our data also indicating that laterality of claw lesion and lying of cattle not having an effect on the occurrence of LDA. Additionally, as etiology of LDA is manifold [1,2,3], numerous factors could have led to relapse in our study population, including, but not only, presence of claw lesions. However, as we did not evaluate the cure rates both in LDA − CLAW and LDA + CLAW after different procedures for the present paper, we cannot make a statement about a possible difference in cure rates as related to claw lesions.

## 5. Conclusions

The results of this retrospective study show that there is no association between laterality of claw lesions and the presence of LDA. Focus should be on the prevention of claw lesions in the post-partum period. Looking at the high number of cattle diagnosed with claw lesions and LDA, diagnostic claw trimming after calving can be recommended, to improve cattle welfare. Additionally, due to the high relapse rates after abomasal rolling, that method should only be considered as a therapy resulting in temporary relief, and surgical intervention should always be discussed.

## Figures and Tables

**Table 1:** Distribution of age and days post-partum of animals diagnosed with left displacement of the abomasum (LDA) and either no claw lesion (LDA − CLAW) or at least one claw lesion (LDA + CLAW). Results with a *p*-value < 0.05 were considered statistically significant and are printed in bold letters. As data was normally distributed, mean and standard deviation (SD) are given.

	Total (*n* = 252)	LDA − CLAW (*n* = 135)		LDA + CLAW (*n* = 117)			Statistic Test
Parameter	Median	Median	IQR ^b^	Median	IQR ^b^	*p*	
Age in years	5.1 (2.0–16.1)	4.6 (2.0–16.1)	2.3	5.5 (2.1–10.4)	2.3	0.001	Mann–Whitney U Test
Days post-partum ^a^	11 (1–86)	10 (1–61)	12	12 (2–86)	12	0.833	Mann–Whitney U Test

^a^ Missing values for *n* = 15 (LDA − CLAW) and *n* = 28 (LDA + CLAW). ^b^ Interquartile Range.

**Table 2:** Laboratory findings of animals diagnosed with left displacement of the abomasum (LDA) and either no claw lesion (LDA − CLAW) or at least one claw lesion (LDA + CLAW). Ranges and physiologic values are given in brackets. Results with a *p*-value < 0.05 were considered statistically significant and are printed in bold letters. Median and Inter-Quartile-Range (IRQ) are given for data which were not normally distributed mean and standard deviation (SD) for data, which were normally distributed. Statistical methods for differently distributed data are indicated.

	Total (*n* = 252)	LDA − CLAW (*n* = 135)		LDA + CLAW (*n* = 117)			Statistic Test
Laboratory Parameter	Median	Median	IQR ^g^	Median	IQR ^g^	*p*	
Betahydroxybutyrate ^a^ (≤1 mmol/L)	1.1 (0–10.8)	1.3 (0–10.8)	2.5	1.0 (0.1–8.97)	1.7	0.155	Mann–Whitney U Test
Glutaraldehyde test ^b^ (≥15 min)	6.5 (0.5–16)	8.0 (0.5–16)	13	4.8 (0.5–16)	14	0.082	Mann–Whitney U Test
Total Protein ^c^ (40–80 g/L)	77.5 (52.6–116.1)	77 (52.6–111.8)	14	79 (56.2–116.1)	11	0.058	Mann–Whitney U Test
Globulin ^d^ (10–40 g/L)	43.1 (24.6–78.6)	41 (24.6–78.6)	14	45 (26.9–78.3)	15	0.005	Mann–Whitney U Test
Leucocyte count ^e^ 4–10 × 10^3^/µL	6.5 (1.6–22)	6.7 (1.6–18.78)	4.1	6.3 (1.9–22)	3.7	0.422	Mann–Whitney U Test
	**Mean**	**Mean**	**SD**	**Mean**	**SD**	***p***	
Albumin ^f^ (30–40 g/L)	33.2 ± 4.5 (19.3–44.5)	34 (23–44.5)	4.4	33 (19.3–43.7)	4.6	0.056	*T*-Test

^a^ Missing values for *n* = 2 (LDA − CLAW). ^b^ Missing values for *n* = 4 (LDA − CLAW) and *n* = 1 (LDA + CLAW) ^c–f^ Missing values for *n* = 1 (LDA − CLAW). ^g^ Interquartile Range.

**Table 3:** Distribution of concurrent diseases in animals diagnosed with left displacement of the abomasum (LDA) and either no claw lesion (LDA − CLAW) or at least one claw lesion (LDA + CLAW). Distribution is given as % of the total number of each LDA − CLAW and LDA + CLAW.

Concurrent Disease	LDA − CLAW (*n* = 135)	LDA + CLAW (*n* = 117)
(Puerperal) metritis	37.8% (*n* = 51)	34.2% (*n* = 40)
Subclinical ketosis	28.9% (*n* = 39)	22.2% (*n* = 26)
Clinical ketosis	24.4% (*n* = 33)	17.9% (*n* = 21)

**Table 4:** Distribution of age (in years) and occurrence of claw trimming in cattle diagnosed with left displacement of the abomasum and no (LDA − CLAW) or at least one (LDA + CLAW) claw lesion. Data about time of last claw trimming was missing for a total number of 35 animals, 19 for LDA − CLAW and 16 for LD + CLAW.

Time of Claw Trimming	Age in Years		
	LDA − CLAW ^a^	LDA + CLAW ^b^	Total ^c^
	*n* = 116	*n* = 101	*n* = 217
Never	2.4 ± 0.3 (2.0–2.9) (*n* = 10)	3.8 ± 2.3 (2.1–7.7) (*n* = 4)	2.8 ± 1.4 (2.0–7.7) (*n* = 14)
<6 months	5.3 ± 2.2 (2.2–16.1) (*n* = 82)	5.5 ± 1.7 (2.4–10.4) (*n* = 79)	5.4 ± 1.7 (2.2–16.1) (*n* = 161)
6 to 12 months	4.5 ± 1.5 (2.5–8.9) (*n* = 21)	5.8 ± 2.0 (2.6–10.3) (*n* = 14)	5.1 ± 1.8 (2.5–10.3) (*n* = 35)
>12 months	5.5 ± 0.7 (4.9–6.2) (*n* = 3)	6.3 ± 2.3 (3.3–8.7) (*n* = 4)	5.9 ± 1.7 (3.3–8.7) (*n* = 7)

^a^ Missing data for *n* = 19; ^b^ Missing data for *n* = 16; ^c^ Missing data for *n* = 35.

**Table 5:** Distribution of occurrence of lameness and an arched back in cattle diagnosed with left displacement of the abomasum and no claw lesion (LDA − CLAW) or at least one claw lesion (LDA + CLAW). ‘Shuffling’ was defined as shuffling with both hind limbs. Results with a *p*-value < 0.05 were considered statistically significant. Significant differences < 0.01 are indicated as **.

	Occurrence of Lameness				Arched Back		
	No	Shuffling	Yes	*p*	No	Yes	*p*
LDA − CLAW (*n* = 135)	50.4% (*n* = 68)	17.0% (*n* = 23)	32.6% (*n* = 44)	**	83.0% (*n* = 112)	17.0% (*n* = 23)	**
LDA + CLAW (*n* = 117)	46.2% (*n* = 54)	17.1% (*n* = 20)	36.8% (*n* = 43)	**	80.3% (*n* = 94)	19.7% (*n* = 23)	**

**Table 6:** Distribution of occurrence of relapse of left displacement of the abomasum (LDA) after rolling in cattle either diagnosed with no (LDA − CLAW) or with (LDA + CLAW) a claw disease, in total, and in animals that received an oral/analgesic treatment. Abomasal rolling was not successful in 5.6% (*n* = 5) of animals of LDA − CLAW, and 5.4% (*n* = 4) of animals in LDA + CLAW. These animals are not included in the table and percentage presented. There was no significant effect of either presence of claw lesion or oral and/or analgesic treatment on occurrence of relapse in cattle with LDA treated by abomasal rolling.

	Relapse (Total) ^a^		Relapse (Following Oral/Analgesic Treatment) ^b^	
	Yes	No	Yes	No
LDA − CLAW	64.4% (*n* = 58)	30.0% (*n* = 27)	68.7% (*n* = 46)	31.3% (*n* = 21)
LDA + CLAW	55.4% (*n* = 41)	39.2% (*n* = 29)	58.9% (*n* = 33)	37.3% (*n* = 23)

^a^ Total number is *n* = 90 for LDA − CLAW, and *n* = 74 for LDA + CLAW. Data about occurrence of relapse was missing for *n* = 1 in LDA − CLAW and *n* = 4 for LDA + CLAW. ^b^ Total number is *n* = 67 for LDA − CLAW, and *n* = 56 for LDA + CLAW.

## Data Availability

The data presented in this study are available on request from the corresponding author. The data are not publicly available due to protection of data privacy of the patients of the Clinic for Ruminants with Ambulatory and Herd Health Services, LMU Munich, Germany.

## Supplementary Materials

The following are available online at https://www.mdpi.com/article/10.3390/ani11061648/s1, Table S1: Listing of analgesics and/or components of oral drench in 169 animals submitted to abomasal rolling for treatment of left displacement of the abomasum at the Clinic for Ruminants with Ambulatory and Herd Health Servicesfrom 2009 to 2019.

## References

[1] LeBlanc S.J., Leslie K.E., Duffield T.F. (2005). Metabolic predictors of displaced abomasum in dairy cattle. J. Dairy Sci..

[2] Van Winden S.C.L., Kuiper R. (2003). Left displacement of the abomasum in dairy cattle: Recent developments in epidemiological and etiological aspects. Vet. Res..

[3] Dirksen G., Doll K., Braun U., Dirksen G., Gründer H.-D., Stöber M. (2006). Krankheiten des labmagens. Innere Medizin und Chirurgie des Rindes.

[4] Rohn M., Tenhagen B.A., Hofmann W. (2004). Survival of dairy cows after surgery to correct abomasal displacement: 1. Clinical and laboratory parameters and overall survival. J. Vet. Med. Ser. A.

[5] Sexton M.F., Buckley W., Ryan E. (2007). A study of 54 cases of left displacement of the abomasum: February to July 2005. Ir. Vet. J..

[6] Sterner K.E., Grymer J., Bartlett P.C., Miekstyn M.J. (2008). Factors influencing the survival of dairy cows after correction of left displaced abomasum. J. Am. Vet. Med. A.

[7] Rohn M., Tenhagen B.A., Hofmann W. (2004). Survival of dairy cows after surgery to correct abomasal displacement: 2. Association of clinical and laboratory parameters with survival in cows with left abomasal displacement. J. Vet. Med. Ser. A.

[8] Zwald N.R., Weigel K.A., Chang Y.M., Welper R.D., Clay J.S. (2004). Genetic selection for health traits using producer-recorded data. II. Genetic correlations, disease probabilities, and relationships with existing traits. J. Dairy Sci..

[9] Nielsen C., Stengärde L., Bergsten C., Emanuelson U. (2013). Relationship between herd-level incidence rate of energy-related postpartum diseases, general risk factors and claw lesions in individual dairy cows recorded at maintenance claw trimming. Acta Vet. Scand..

[10] Gomez A., Cook N.B. (2010). Time budgets of lactating dairy cattle in commercial freestall herds. J. Dairy Sci..

[11] González L.A., Tolkamp B.J., Coffey M.P., Ferret A., Kyriazakis I. (2008). Changes in feeding behavior as possible indicators for the automatic monitoring of health disorders in dairy cows. J. Dairy Sci..

[12] Smith B.I., Kauffold J., Sherman L. (2010). Serum haptoglobin concentrations in dairy cattle with lameness due to claw disorders. Vet. J..

[13] O’Driscoll K., McCabe M., Earley B. (2015). Differences in leukocyte profile, gene expression, and metabolite status of dairy cows with or without sole ulcers. J. Dairy Sci..

[14] Ito K., Von Keyserlingk M.A.G., LeBlanc S.J., Weary D.M. (2010). Lying behavior as an indicator of lameness in dairy cows. J. Dairy Sci..

[15] Yunta C., Guasch I., Bach A. (2012). Lying behavior of lactating dairy cows is influenced by lameness especially around feeding time. J. Dairy Sci..

[16] Eberhart N.L., Krawczel P.D. (2017). The effect of hock injury laterality and lameness on lying behaviors and lying laterality in holstein dairy cows. Animals.

[17] Feist M., Köstlin R., Nuss K. (2008). Claw surgery in cattle: The benefit of perioperative analgesics. Tierarztl. Prax. Ausgabe G.

[18] Zadnik T., Lombar R. (2011). Our experience with left-sided abomasal displacement correction via the roll-and-toggle-pin suture procedure according to Grymer/Sterner model. ISRN Vet. Sci..

[19] Hädrich G. (2007). Untersuchungen zu der Entwicklung der Körperkondition, dem Peripartalen Stoffwechsel und der Morbidität von Hochleistungskühen. Ph.D. Thesis.

[20] Opsomer G., Laurier L., de Kruif A., Murray P.D. (1998). Left displaced abomasum: Considerations of treatment method and a case report of mesenteric torsion after rolling. Vet. Q..

[21] Sandholm M. (1974). Die Feststellung der hyper-γ-Globulinämie beim rind unter Praxisbedingungen. Tierärztl. Prax..

[22] Metzner M., Horber J., Rademacher G., Klee W. (2007). Application of the glutaraldehyde test in cattle. J. Vet. Med. Ser. A.

[23] Oetzel G.R. (2004). Monitoring and testing dairy herds for metabolic disease. Vet. Clin. N. Am. Food A.

[24] Janowitz H. (1998). Laparoskopische reposition und fixation des nach links verlagerten labmagens beim rind. Tierärztl. Prax..

[25] Dirksen G. (1967). Gegenwärtiger stand der diagnostik, therapie und prophylaxe der dislocatio abomasi sinistra des rindes. Dtsch. Tieraerztl. Wochenschr..

[26] Greenough P.R., Greenough P.R. (2007). Introduction. Bovine Laminitis and Lameness.

[27] Walker S.L., Smith R.F., Routly J.E., Jones D.N., Morris M.J., Dobson H. (2008). Lameness, activity time-budgets, and estrus expression in dairy cattle. J. Dairy Sci..

[28] Newcomer B.W., Chamorro M.F. (2016). Distribution of lameness lesions in beef cattle: A retrospective analysis of 745 cases. Can. Vet. J..

[29] Fiedler A., Maierl J., Nuss K., Fiedler A., Maierl J., Nuss K. (2019). Funktionelle Klauenpflege. Erkrankungen der Klauen und Zehen des Rindes.

[30] Becker J., Steiner A., Kohler S., Koller-Bähler A., Wüthrich M., Reist M. (2014). Lameness and foot lesions in Swiss dairy cows: I. Prevalence. Schweiz. Arch. Tierheilkd..

[31] Kofler J., Hangl A., Pesenhofer R., Landl G. (2011). Evaluation of claw health in heifers in seven dairy farms using a digital claw trimming protocol and claw data analysis system. Berl. Munch. Tierarztl. Wochenschr..

[32] Shearer J.K., Plummer P.J., Schleining J.A. (2015). Perspectives on the treatment of claw lesions in cattle. Vet. Med. Res. Rep..

[33] Huxley J., Archer S., Bell N., Brunell M., Green L., Potterton S., Reader J., Green M. (2012). Control of lameness. Dairy Herd Health.

[34] Shearer J.K., van Amstel S.R. (2017). Pathogenesis and treatment of sole ulcers and white line disease. Vet. Clin. N. Am. Food A.

[35] Solano L., Barkema H.W., Pajor E.A., Mason S., LeBlanc S.J., Zafffino Heyerhoff J.C., Nash C.G.R., Haley D.B., Vasseur E., Pellerin D. (2015). Prevalence of lameness and associated risk factors in Canadian Holstein-Friesian cows housed in freestall barns. J. Dairy Sci..

[36] Machado V.S., Caixeta L.S., Bicalho R.C. (2011). Use of data collected at cessation of lactation to predict incidence of sole ulcers and white line disease during the subsequent lactation in dairy cows. Am. J. Vet. Res..

[37] Fjeldaas T., Nafstad O., Fredriksen B., Ringdal G., Sogstad Å.M. (2007). Claw and limb disorders in 12 Norwegian beef-cow herds. Acta Vet. Scand..

[38] Tadich N., Flor E., Green L. (2010). Associations between hoof lesions and locomotion score in 1098 unsound dairy cows. Vet. J..

[39] Navarro G., Green L.E., Tadich N. (2013). Effect of lameness and lesion specific causes of lameness on time budgets of dairy cows at pasture and when housed. Vet. J..

[40] Manske T., Hultgren J., Bergsten C. (2002). The effect of claw trimming on the hoof health of Swedish dairy cattle. Prev. Vet. Med..

[41] Mahendran S., Bell N. (2015). Lameness in cattle 2. Managing claw health through appropriate trimming techniques. In Practice.

[42] Thomsen P.T., Foldager L., Raundal P., Capion N. (2019). Lower odds of sole ulcers in the following lactation in dairy cows that received hoof trimming around drying off. Vet. J..

[43] Griffiths B.E., Dai White G., Oikonomou G. (2018). A cross-sectional study into the prevalence of dairy cattle lameness and associated herd-level risk factors in England and Wales. Front. Vet. Sci..

[44] Somers J.R., Huxley J.N., Doherty M.L., O’Grady L.E. (2019). Routine herd health data as cow-based risk factors associated with lameness in pasture-based, spring calving irish dairy cows. Animals.

[45] Jewell M.T., Cameron M., Spears J., McKenna S.L., Cockram M.S., Sanchez J., Keefe G.P. (2019). Prevalence of lameness and associated risk factors on dairy farms in the Maritime Provinces of Canada. J. Dairy Sci..

[46] Barragan A.A., Pineiro J.M., Schuenemann G.M., Rajala-Schultz P.J., Sanders D.E., Lakritz J., Bas S. (2018). Assessment of daily activity patterns and biomarkers of pain, inflammation, and stress in lactating dairy cows diagnosed with clinical metritis. J. Dairy Sci..

[47] Pohl A., Burfeind O., Heuwieser W. (2015). The associations between postpartum serum haptoglobin concentration and metabolic status, calving difficulties, retained fetal membranes, and metritis. J. Dairy Sci..

[48] Newman K.D., Harvey D., Roy J.-P. (2008). Minimally invasive field abomasopexy techniques for correction and fixation of left displacement of the abomasum in dairy cows. Vet. Clin. N. Am. Food A.

[49] Mueller K. (2011). Diagnosis, treatment and control of left displaced abomasum in cattle. InPractice.

[50] Niehaus A.J. (2008). Surgery of the abomasum. Vet. Clin. N. Am. Food A.

[51] Trent A.M., Fubini D.L., Ducharme N.G. (2017). Surgery of the abomasum. Farm Animal Surgery.

[52] González-Martín J.V., Pérez-Villalobos N., Baumgartner W., Astiz S. (2019). An investigation into the development of right displaced abomasum by rolling 268 dairy cows with left displaced abomasum. J. Dairy Sci..

[53] Lombar R., Zadnik T. Conservative treatment of left-side displacement of the abomasum (LDA) with rolling technique and oral electrolyte therapy. Proceedings of the XXVI World Buiatrics Conference.

