# Supplementary material for: Retrospective Evaluation of Claw Lesions, Inflammatory Markers, and Outcome after Abomasal Rolling in Cattle with Left Displacement of the Abomasum
Source: Animals (Basel). 2021 Jun 1;11(6):1648. doi: 10.3390/ani11061648 (PMC8229859; doi:10.3390/ani11061648)
Supplement: Supplementary file 1 [file animals-11-01648-s001.zip › animals-1238290-supplementary.pdf]

Table S1: Listing of analgesics and/or components of oral drench in 169 animals submitted to abomasal rolling for treatment of left displacement of the abomasum at the Clinic for Ruminants with Ambulatory and Herd Health Services from 2009 to 2019. Due to the retrospective nature of the manuscript, animals were treated differently within the study population. Number of animals is abbreviated as Animal.

Animal	Analgesic treatment	Oral Drench
1	- None	- Propylene glycol<br>- Sodium Sulfate<br>- Vitamin E/Selenium<br>- KCl
2	- Flunixine meglumine	- None
3	- Ketoprofen	- None
4	- Metamizole	- None
5	- Flunixine meglumine	- Propylene glycol<br>- Vitamin E/Selenium<br>- Ca
6	- Meloxicam	- None
7	- Flunixine meglumine<br>- Metamizol	- Propylene glycol<br>- Vitamin E/Selenium<br>- KCl
8	- Meloxicam	- None
9	- Meloxicam<br>- Melosolute	- None
10	- None	- Sodium sulfate<br>- Vitamin E/Selenium<br>- KCl<br>- Ca
11	- Ketoprofen	- Propylene glycol<br>- KCl<br>- Ca
12	- Flunixine meglumine<br>- Metamizole	- Propylene glycol<br>- Vitamin E/Selenium<br>- KCl<br>- Ca
13	- Flunixine meglumine	- Propylene glycol<br>- Vitamin E/Selenium<br>- KCl<br>- Ca
14	- Metamizole	- None
15	- Flunixine meglumine<br>- Metamizol	-
16	- Ketoprofen	- Propylene glycol<br>- Vitamin E/Selenium<br>- KCl<br>- Ca
17	- Meloxicam	- KCl
18	- Meloxicam	- None
19	- Ketoprofen	- None
20	- Ketoprofen	- None
21	- Metamizole	- None
22	- Flunixine meglumine<br>- Metamizole	- None

Animal	Analgesic treatment	Oral Drench
23	- Ketoprofen	- Propylene glycol<br>- Sodium Sulfate<br>- Vitamin E/Selenium<br>- KCl<br>- Ca
24	- Meloxicam	- None
25	- Flunixine meglumine	- None
26	- Meloxicam	- Propylene glycol<br>- Vitamin E/Selenium<br>- KCl<br>- Ca
27	- Meloxicam	- None
28	- Flunixine meglumine	- None
29	- Flunixine meglumine<br>- Metamizole	- Propylene glycol<br>- Sodium Sulfate<br>- Vitamin E/Selenium<br>- KCl
30	- Meloxicam	- None
31	- Flunixine meglumine	- None
32	- Flunixine meglumine	- Propylene glycol<br>- Vitamin E/Selenium<br>- NadPh
33	- Meloxicam	- None
34	- Ketoprofen	- Propylene glycol<br>- KCl<br>- Ca
35	- Meloxicam	- KCl<br>- NaCl
36	- Flunixine meglumine	- Sodium Sulfate<br>- Vitamin E/Selenium<br>- KCl
37	- Ketoprofen<br>- Metamizole	- Sodium Sulfate<br>- Vitamin E/Selenium<br>- KCl<br>- Ca<br>- NaCl
38	- None	- None
39	- Meloxicam	- Propylene glycol<br>- KCl<br>- Ca
40	- Ketoprofen	- Propylene glycol<br>- Vitamin E/Selenium<br>- NadPh
41	- Flunixine meglumine	- Propylene glycol<br>- Vitamin E/Selenium<br>- KCl
42	- Flunixine meglumine	- Propylene glycol<br>- Vitamin E/Selenium<br>- KCl<br>- Ca

Animal	Analgesic treatment	Oral Drench
43	- Flunixine meglumine	- Propylene glycol<br>- Sodium Sulfate<br>- Vitamin E/Selenium<br>- KCl
44	- Flunixine meglumine<br>- Metamizole	- Proylene glycol<br>- Vitamin E/Selenium<br>- KCl
45	- Dexamethasone	- Propylene glycol<br>- KCl
46	- Ketoprofen	- None
47	- Ketoprofen	- None
48	- Flunixine meglumine	- Propylene glycol<br>- Vitamin E/Selenium<br>- KCl
49	- Ketoprofen	- Propylene glycol<br>- Vitamin E/Selenium<br>- NaCl<br>- NaBic
50	- Ketoprofen	- Propylene glycol<br>- Vitamin E/Selenium<br>- KCl<br>- Ca
51	- Flunixine meglumine	- Propylene glycol<br>- KCl<br>- Ca
52	- Flunixine meglumine	- Vitamin E/Selenium<br>- KCl<br>- NadPh
53	- Flunixine meglumine	- Vitamin E/Selenium<br>- KCl
54	- Ketoprofen	- Propylene glycol<br>- Sodium Sulfate<br>- KCl<br>- Ca<br>- NaBic
55	- Ketoprofen	- Sodium Sulfate<br>- Vitamin E/Selenium<br>- KCl<br>- Ca
56	- Flunixine meglumine	- None
57	- None	- Propylene glycol<br>- Vitamin E/Selenium<br>- NadPh
58	- None	- None
59	- Flunixine meglumine	- None
60	- Ketoprofen	- Propylene glycole<br>- Sodium Sulfate<br>- Vitamin E/Selenium<br>- KCl<br>- Ca

Animal	Analgesic treatment	Oral Drench
61	- Flunixine meglumine	- Propylene glycol<br>- Sodium Sulfate<br>- KCl<br>- Ca<br>- NaBic
62	- Flunixine meglumine	- Sodium Sulfate<br>- KCl<br>- Ca<br>- NaBic
63	- Ketoprofen	- Propylene glycol<br>- Vitamin E/Selenium<br>- KCl<br>- Ca
64	- Flunixine meglumine	- Vitamin E/Selenium<br>- KCl<br>- Ca
65	- Flunixine meglumine	- Vitamin E/Selenium<br>- KCl
66	- Flunixine meglumine	- None
67	- Flunixine meglumine	- None
68	- Flunixine meglumine	- Propylene glycol<br>- Vitamin E/Selenium<br>- KCl
69	- Flunixine meglumine	- Propylene glycol<br>- Vitamin E/Selenium<br>- KCl<br>- Ca
70	- Flunixine meglumine	- Vitamin E/Selenium<br>- KCl<br>- Ca
71	- Ketoprofen	- None
72	- Flunixine meglumine	- None
73	- Flunixine meglumine<br>- Metamizole	- Propylene glycol<br>- Vitamin E/Selenium<br>- KCl<br>- Ca
74	- Flunixine meglumine	- Propylene glycol<br>- Sodium Sulfate<br>- Vitamin E/Selenium
75	- Flunixine meglumine	- None
76	- Flunixine meglumine	- Propylene glycol<br>- Sodium Sulfate<br>- Vitamin E/Selenium<br>- KCl
77	- Meloxicam	- None
78	- None	- None
79	- Ketoprofen	- Propylene glycol<br>- Sodium Sulfate<br>- KCl<br>- Ca
80	- Ketoprofen	- None

Animal	Analgesic treatment	Oral Drench
81	- Flunixine meglumine	- None
82	- Flunxine meglumine	- Sodium Sulfate<br>- Vitamin E/Selenium<br>- KCl
83	- Flunixine meglumine	- None
84	- None	- None
85	- Flunixine meglumine	- Sodium Sulfate<br>- Vitamin E/Selenium<br>- KCl
86	- Flunixine meglumine	- Vitamin E/Selenium<br>- NadPh
87	- Flunixine meglumine	- Propylene glycol<br>- Vitamin E/Selenium<br>- KCl<br>- NadPh
88	- Flunixine meglumine	- Propylene glycol<br>- Sodium Sulfate<br>- Vitamin E/Selenium<br>- KCl<br>- NadPh
89	- Ketoprofen	- None
90	- Flunixine meglumine	- Propylene glycol<br>- Vitamin E/Selenium<br>- KCl
91	- Flunixine meglumine	- Propylene glycol<br>- Vitamin E/Selenium<br>- KCl<br>- Ca
92	- Meloxicam	- None
93	- None	- Vitamin E/Selenium<br>- KCl<br>- Ca
94	- Flunixine meglumine	- None
95	- Ketoprofen	- Vitamin E/Selenium<br>- Ca
95	- None	- None
97	- Ketoprofen	- None
98	- Flunixine meglumine	- Propylene glycol<br>- Vitamin E/Selenium<br>- KCl<br>- Ca
99	- Flunixine meglumine	- Propylene glycol<br>- Vitamin E/Selenium<br>- KCl<br>- NaBic<br>- MgO
100	- None	- Vitamin E/Selenium<br>- KCl<br>- Ca
101	- Ketoprofen	- None
102	- None	- None

Animal	Analgesic treatment	Oral Drench
103	- Ketoprofen	- None
104	- Flunixine meglumine	- Propylene glycol<br>- Vitamin E/Selenium<br>- KCl<br>- Ca
105	- Meloxicam	- None
106	- Ketoprofen	- None
107	- Ketoprofen	- None
108	- Ketofprofen	- Sodium Sulfate<br>- Vitamin E/Selenium<br>- KCl
109	- Flunixine meglumine	- Vitamin E/Selenium<br>- KCl<br>- Ca
110	- Flunixine meglumine	- Propylene glycol<br>- KCl<br>- Ca
112	- Flunixine meglumine	- None
112	- Flunixine meglumine	- None
113	- Flunixine meglumine	- Propylene glycol<br>- Vitamin E/Selenium<br>- KCl<br>- Ca
114	- None	- None
115	- Flunixine meglumine	- None
116	- Meloxicam	- None
117	- Flunixine meglumine	- Propylene glycol<br>- Vitamin E/Selenium<br>- KCl
118	- Meloxicam	- None
119	- Ketoprofen	- None
120	- Meloxicam	- None
121	- Meloxicam	- None
122	- Flunixine meglumine	- None
123	- Flunixine meglumine	- None
124	- Flunixine meglumine	- None
125	- Ketoprofen	- None
126	- Flunixine meglumine	- None
127	- Meloxicam	- None
128	- Flunixine meglumine	- None
129	- None	- None
130	- Ketoprofen	- None
131	- Flunixine meglumine	- Propylene glycol<br>- Sodium Sulfate<br>- Vitamin E/Selenium<br>- KCl<br>- Ca
132	- Meloxicam	- None
133	- Flunixine meglumine	- Vitamin E/Selenium<br>- KCl

Animal	Analgesic treatment	Oral Drench
134	- Ketoprofen	- None
135	- Meloxicam	- Vitamin E/Selenium<br>- Ca
136	- Ketoprofen	- None
137	- Flunixine meglumine	- None
138	- Flunixine meglumine	- None
139	- None	- None
140	- Meloxicam	- None
141	- Flunixine meglumine	- None
142	- None	- None
143	- Ketoprofen	- None
144	- Flunixine meglumine	- None
145	- Flunixine meglumine	- None
146	- None	- Propylene glycol<br>- Vitamin E/Selenium<br>- KCl<br>- Ca
147	- None	- None
148	- Ketoprofen	- None
149	- None	- None
150	- None	- None
151	- None	- None
152	- Flunixine meglumine	- None
153	- Ketoprofen	- None
154	- Flunixine meglumine	- Propylene glycol<br>- Sodium Sulfate<br>- Vitamin E/Selenium<br>- KCl
155	- Ketoprofen	- None
154	- None	- None
157	- Flunixine meglumine	- None
158	- None	- None
159	- Flunixine meglumine	- None
160	- None	- None
161	- Ketoprofen	- None
162	- Ketoprofen	- None
163	- Flunixine meglumine	- None
164	- Flunixine meglumine	- None
165	- Ketoprofen	- None
166	- None	- None
167	- None	- None
168	- Flunixine meglumine	- None
169	- None	- None
